# Exploring the Influence of Different *Saccharomyces cerevisiae* Strains and Hop Varieties on Beer Composition and Sensory Profiles

**DOI:** 10.3390/foods14132357

**Published:** 2025-07-02

**Authors:** Antonella Costantini, Maurizio Petrozziello, Christos Tsolakis, Andriani Asproudi, Enrico Vaudano, Laura Pulcini, Federica Bonello, Katya Carbone, Maria Carla Cravero

**Affiliations:** 1 Council for Agricultural Research and Economics—Research Centre for Viticulture and Enology (CREA-VE), Via P. Micca 35, 14100 Asti, Italy; 2 Council for Agricultural Research and Economics—Research Centre for Olive, Fruit and Citrus Crops (CREA-OFA), Via di Fioranello 52, 00134 Rome, Italy

**Keywords:** oenological *Saccharomyces cerevisiae*, *IRC7* gene, hop, polyfunctional thiols, beer, volatile compounds, sensory analysis

## Abstract

The influence of different *Saccharomyces cerevisiae* (*Sc*) strains and hop varieties on the physical, chemical and sensory properties of beer was investigated. ISE77, an oenological *Sc* strain screened for the *IRC7* gene and β-lyase activity, and a commercial yeast, as a control, were experimented with two hops (dry hopping), Mosaic^®^ (M) and Hallertau Mittelfrüh (HM). Both hop variety and yeast strain exerted a considerable influence on the organoleptic profile of the beer. Samples with M hops exhibited elevated levels of specific volatile compounds (e.g., limonene and linalool). ISE77 generated higher levels of esters, irrespective of the hop variety employed, imparting fruity and floral characteristics. Moreover, the beers fermented with ISE77 showed herbal and spicy notes. Regardless of the hop variety, samples brewed with the control yeast showed higher honey and caramel note levels. Beers fermented with ISE77 and HM exhibited remarkable similarities to those produced with ISE77 and M, particularly for some odour attributes (citrus, exotic fruits, and aromatic herbs). These attributes were more intense than in beers fermented with the control yeast and HM. This study demonstrated the potential of oenological *Sc* strains to achieve innovative brewing outcomes when combined with selected hops.

## 1. Introduction

The growth and popularity of the craft brewing sector can be attributed to its focus on diversification and regional identity [1].

Craft beer is a special segment of the beverage industry that attracts beer enthusiasts who value its distinct characteristics, explaining its evolution. Unlike traditional industrial beer production, microbreweries promote the exploration of diverse beer styles [2]. Small breweries cater to consumers seeking beers with distinctive profiles, crafted from local raw materials, and produced by master brewers with exceptional expertise in beer culture. According to Jaeger et al. [3], craft beer drinkers are typically distinguished from traditional beer consumers based on their preference for innovative beers that have novel and complex sensory profiles. A close analysis of the consumption of craft beer shows that these types of beers are usually chosen because of their variety in flavour, which again makes them be perceived as superior quality compared to commercial beer [4].

The main actors in beer are water, malt, hops, and yeasts; these last represent a source of flavour diversification.

As a natural consequence, research increasingly turns to yeast experimentation to create unique aromas and flavours that define beers. In beer production, yeasts play a crucial role, significantly influencing the beer’s characteristics and quality. Various yeast strains and species predominantly determine fermented drinks’ intricate nature and sensory aspects [5]. Therefore, the fermentation stage offers the greatest opportunity for beer differentiation.

Among yeasts, *Saccharomyces cerevisiae* is the dominant microbial actor and already has a widespread industrial role [6], being responsible for the production of aromatic compounds during alcoholic fermentation and strongly influencing the sensory properties of the final products [7,8] due to its secondary metabolism, which is highly adapted to specific fermentation conditions and substrates in terms of temperature and sugar composition, among others [9]. In this regard, using yeast starter strains of oenological origin as brewing starters has been the focus of much effort in recent years [1,10,11].

After yeast fermentation, hops are considered the most critical component affecting beer quality. There are many hop varieties on the market, and approximately 97% of the total crop is used in the brewing process, providing the characteristic bitterness and contributing to the aroma profile of the beverage [12,13], as well as its microbiological stability due to its antiseptic properties [14]. Commercial hop varieties can be classified as “bittering hops”, “aroma hops”, and “dual-purpose hops” based on their chemical composition in relation to their use in brewing. Aroma hops usually have a low alpha-acid concentration (<5%) and a high concentration of aroma oils, whereas bitter hops are characterised by a high alpha-acid content (>5%; co-humulone <30%). The so-called dual-purpose hops are intended to add hop aroma to beer but also contain high levels of alpha acids that make the beer bitter [15]. However, in recent years, especially in the craft brewing sector, in addition to the content of bitter acids and essential oils, the contribution of some thiols present in the hops (free polyfunctional thiols) to the final bouquet of the beer and the potential contribution of their bound odourless precursors have become increasingly important [16]. Despite the presence of minimal amounts of sulphur compounds in hops, these substances can significantly affect the overall beer flavour. Many of them are considered off-flavours [17]; however, polyfunctional thiols are highly desirable, contributing fruity, citrus, tropical fruit, or black and white currant notes. Due to their low perception thresholds (a few nanograms per litre), they contribute significantly to the aroma profile of several beverages, including beer [18].

Varietal thiols have long been recognised as significant contributors to the overall quality of wine; however, their presence in both beer and hops was only demonstrated by researchers starting in the early 2000s [19]. The primary aromatic volatile thiol is 4-methyl-4-sulfanylpentan-2-one, also referred to as 4-methyl-4-mercaptopentan-2-one (4MMP). This compound is a potent aroma contributor with “black currant” and “fruity” odour descriptors, detectable at extremely low concentrations due to its remarkably low odour threshold of approximately 1.5 ng/L [20]. Additionally, the characteristic notes of passion fruit and grapefruit in beer are attributed to 3-sulfanylhexan-1-ol, also named 3-mercaptohexan-1-ol (3MH) [21,22]. Furthermore, its acetate derivative, 3-sulfanylhexyl acetate or 3-mercaptohexan-1-ol acetate (3MHA), is another key thiol that significantly contributes to the citrus aroma of some beers [23].

Recent studies have extensively investigated the composition of hops, highlighting the relevant presence of polyfunctional thiols. The hop variety Nelson Sauvin is renowned for its distinct thiolic aroma, primarily due to 3-mercapto-4-methylpentan-1-ol, another polyfunctional thiol found in hops, which imparts “exotic fruit”, “rhubarb-like”, and “grapefruit” notes to the beer [24]. This compound is also significant in Hallertau Blanc and Tomahawk hops, with higher levels observed in dry-hopped beers [16]. Other hops, such as Citra, Simcoe, Summit, and Cascade, contain relevant quantities of 4MMP [16]. Additionally, Hallertau Blanc, Comet, and Tomahawk hops have high levels of 3MH. In this study, we utilised Mosaic^®^ hops, which exhibit very high concentrations of both 4MMP and 3MH compared to other hop varieties [18].

Although research on this topic is ongoing and information on the interaction between thiols and other aromatic compounds is limited, it is plausible to hypothesise that polyfunctional thiols interact synergistically with other aromatic compounds, enhancing the complexity and overall perception of the aromatic bouquet. For instance, the combination of thiols with hop-derived monoterpenoids may intensify citrus notes, resulting in distinctive and pronounced flavour profiles

Within grape must, these thiols are predominantly found in non-volatile forms, bound to amino acids or small peptides, and they are released during the fermentation phase by the action of yeasts (e.g., glutathione and cysteine) [19,21,22,25]. It follows that the final content of aromatic thiols in wine is closely linked to the ability of fermentation yeasts to metabolise them through the action of endogenous enzymes with β-lyase activity [26]. The Irc7p enzyme is one of the main β-lyase involved in the conversion of Cys-precursor into thiol [27]; according to Belda et al. [28], the gene coding for this protein can be present in a longer form or a short form, depending on the strain; the long form shows a fully active enzyme, highlighting the dependence of the release of aromatic thiols on the yeast strain.

Research has shown that, in hops, the glutathionylated conjugate is the predominant form of 3MH. In contrast, for 4MMP, both free and bound forms appeared to be equally significant, with each contributing to the presence of 4MMP in beer. Recently, Cys- and G-sulfanyl alkyl aldehydes and acetates were also found in hops [29].

As with grapes, the presence and concentration of functional thiols in hops mainly depend on their variety [30,31]. Consequently, in recent years, there has been significant interest from both the scientific community and the brewing industry in the biotransformation of hop compounds during the brewing process.

In the present study, sixty oenological *S. cerevisiae* strains were screened by PCR targeted on the *IRC7* gene. One of the strains with the long form of this gene was then used in wort fermentation and compared to a commercial strain normally used for brewing, in the presence of two different hop varieties. The goal was to assess how yeast and its interaction with hop can affect the final products in terms of chemical composition, volatile compounds, and sensory profile.

## 2. Materials and Methods

### 2.1. Yeast Strains

Fifty-seven *Saccharomyces cerevisiae* strains belonging to the culture collection of oenological and viticultural environment (CREA-CMVE) of CREA-VE, Asti, and three oenological commercial starters (TXL, FTH, and STR, Lamothe-Italia, Verona, Italy) were employed in this study.

### 2.2. PCR Screening for β-Lyase Gene

Yeasts were grown in YEPG (1% yeast extract, 1% peptone, and 2% glucose) at 25 °C. DNA was extracted according to [32]. PCR was conducted using the primers PF6, 5′-AGCTGGTCTGGAGAAAATGG-3′ and PR7, 5′-TCTTCTGCGAGACGTTCAAA-3′ with the following conditions: an initial denaturing step of 2 min at 94 °C, followed by 35 cycles of 94 °C for 15 s, 56 °C for 30 s, and 72 °C for 1 min, and then a final extension at 72 °C for 5 min, as described by [28]. Amplicons were run on 2% agarose gels to verify the size of the amplified products. Images were visualised after staining with ethidium bromide using GelDoc (Bio-Rad, Milan, Italy).

### 2.3. Choice of the Strain and Evaluation of β-Lyase Activity

The PCR assays were the first step of this selection. After identifying the strains with the long form of the β-lyase gene, further tests were conducted to find a strain with good β-lyase activity. The first was a colourimetric qualitative test, in which agar plates containing the medium described by [28] were used, supplemented with agar (1.5%) and bismuth. In the second test, a preliminary semiquantitative trial was conducted using a specific culture medium assay, followed by gas chromatographic analysis [28]. This approach involved measuring the production of methyl sulfide and dimethyl disulfide in the yeast growth medium supplemented with S-methylcysteine, an amino acid specifically degraded by β-lyases to form these compounds. The medium was inoculated with the selected yeast strain at 1% and sampled after 7 days. A 10 mL aliquot of the sample was prepared by adding 2 g of NaCl and 100 µL of an internal standard solution (1-heptanol), then diluted with ultrapure water to a final volume of 20 mL. The sample was extracted using solid phase micro extraction (SPME) and analysed by GC-MS according to the method described by [33].

### 2.4. Yeast Strains and Preparation of the Starters

As a result of the yeast selection phases, the *S. cerevisiae* ISE 77 strain, belonging to the CREA-CMVE, was employed in fermentation trials. The commercial strain Rock (Brewline, Italy) was used as a control. Both yeasts were precultured in YEPG medium (1% yeast extract, 1% peptone, and 2% glucose) and incubated at 25 °C with shaking for two days. They were then propagated in YEPG for 3 days at 25 °C with shaking. The inoculum size was 5 × 10^6^ cells/mL.

### 2.5. Hops

Hop pellets (T90) of Hallertau Mittelfrüh (HM; α-acids: 3.8%; β-acids: 4%; cohumulone: 22%; crop year: 2019; total oils: 1%; purpose: aroma hops) and Mosaic^®^ (M; α-acids: 11.3%; β-acids: 3.6%; cohumulone: 25%; crop year: 2019; total oils: 2%; purpose: dual-purpose hops) varieties were purchased from Birramia (Querceta, Italy). Hops were added at the beginning of fermentation (2.5 g/L) and after 2/3 of fermentation (2.5 g/L).

### 2.6. Fermentation

Small-scale 4L fermentation trials at 22 °C with three replicates (A, B, and C) were carried out. The vessels used in this experiment were 5-litre glass demijohns with rounded bodies and narrow necks, sealed with fermentation locks. The wort was prepared using Beermalt Liquid LIGHT—Liquid Barley Malt Extract (Birramia, Italy), according to the manufacturer’s instructions. The unhopped wort extract was diluted with water to 10.5 °Brix/1.040 SG. The experimental design is shown in Table 1.

Fermentation kinetics were evaluated by measuring the weight loss until a constant weight was obtained. At the end of the fermentations, the samples were bottled for further analyses.

### 2.7. Chemical–Physical Analyses

Volatile and total acidity were analysed at the end of fermentation according to the OIV Methods. Ethanol content and final density were determined, after distillation, using a densimeter (Anton Paar, DMA 4500M, Graz, Austria). The pH was measured using potentiometry with a PHM240 precision pH meter (Radiometer Analytical, Copenhagen, Denmark).

### 2.8. Analysis of the Volatile Aromatic Fraction by GC-MS After SPME Extraction

Solid-phase microextraction (SPME) was employed as a solvent-free method for extracting volatile organic compounds (VOCs). A 50/30 µm SPME fibre assembly, Divinylbenzene/Carboxen/Polydimethylsiloxane (DVB/CAR/PDMS) (Supelco, Bellefonte, PA, USA), was used due to its high efficiency in adsorbing a wide range of volatile compounds. For each sample, 5 mL of liquid was diluted 1:2 with distilled water to achieve a final ethanol concentration of approximately 2.5% *v*/*v*. A total of 5 µL of the internal standard, ethyl heptanoate, was added to the sample. Then, to enhance extraction efficiency, 2 g of sodium chloride (NaCl) was added to the diluted samples to increase ionic strength and promote the “salting-out” effect, facilitating the release of volatile compounds. Additionally, 1% *w/v* ethylenediaminetetraacetic acid (EDTA) was included as a chelating agent to bind metal ions, reducing interference and improving extraction sensitivity.

The prepared sample vial was placed into a heated autosampler tray (GERSTEL GmbH & Co. KG, Mülheim an der Ruhr, Germany). The SPME fibre was exposed to the sample headspace at 35 °C for 30 min, allowing sufficient time for the adsorption of volatile compounds.

GC-MS analysis was performed using an Agilent 6890 Series gas chromatograph coupled with an Agilent 5975C Mass Selective Detector (Agilent Technologies, Santa Clara, CA, USA). After extraction, the SPME fibre was thermally desorbed in the GC injection port at 280 °C under splitless conditions, releasing the adsorbed volatile compounds into the system.

Chromatographic separation was achieved using a Restek Rxi-5ms capillary column (30 m × 0.25 mm × 0.25 µm, Restek Corporation, Bellefonte, PA, USA) to ensure high-resolution analysis. The helium carrier gas was maintained at a constant flow rate of 1.1 mL/min. The oven temperature program was as follows: an initial temperature of 40 °C was held for 5 min, followed by a ramp at 2.00 °C/min to 100 °C, with no hold, and a second ramp at 7.00 °C/min to a final temperature of 270 °C, which was held for 1 min. The total run time was 60.29 min, with an equilibration time of 0.50 min between runs.

The mass spectrometer operated in electron impact (EI) mode at 70 eV, and data were acquired in Total Ion Current (TIC) mode over a mass range of 35–350 *m*/*z* at a rate of 20 Hz. The MS detector was operated with a transfer line temperature set to 230 °C and a quadrupole temperature of 150 °C. Compound identification was conducted by comparing the mass spectra of the analytes with the NIST 14 (National Institute of Standards and Technology, Gaithersburg, MD, USA) and Wiley 275 spectral libraries (John Wiley & Sons, Hoboken, NJ, USA). A minimum match quality threshold of 80% was used to ensure reliable identification. Linear retention indices (LRI) were calculated using the Van den Dool and Kratz method, which relies on the retention times of the analytes and a homologous series of n-alkanes (C8–C40) injected under identical chromatographic conditions. Quantitative analysis was performed using ethyl heptanoate as an internal standard, which was added to each sample prior to analysis. Quantifications were normalised to the internal standard, which was added to each sample prior to analysis. Results were expressed as µg/L internal standard equivalents.

### 2.9. Analysis of Polyfunctional Thiols

The thiols were analysed using SPE/GC-MS after derivatisation, adapting the method proposed by Herbst-Johnstone et al. [34]. Briefly, polyfunctional thiols (3-mercaptohexanol, 4-mercapto-4-methyl-2-pentanone, and 3-mercaptohexylacetate) were derivatised using ethylpropiolate in an alkaline medium (pH 10), then extracted with a reversed-phase SPE cartridge (C18 EC, 1 g, Biotage, Sweden). Derivative compounds were separated on a non-polar column (Restek Superchrom, Rxi-5ms, 30 × 0.25 × 0.25) using an Agilent 6890 Series gas chromatograph coupled with an Agilent 5975C Mass Selective Detector (Agilent Technologies, Santa Clara, CA, USA). Mass spectra for thiol derivatives were recorded by acquiring signals in Single Ion Monitoring (SIM) mode to improve sensitivity. The monitored ions and their retention times were as follows (the highlighted ion was used for quantification): 4-MMP-ETP (retention time: 21.93 min, ions: 230, 132), 3-MHA-ETP (retention time: 27.32 min, ions: 229, 274), 3-MH-ETP (retention time: 25.53 min, ions: 187, 232), and 6-ME-ETP (retention time: 28.24 min, ions: 187, 232). The electronic ionisation (EI) source was set at 230 °C with a potential of 70 eV, and the quadrupole temperature was maintained at 150 °C.

### 2.10. Sensory Analysis

The sensory analyses were carried out in the sensory laboratory of CREA-VE in Asti following ISO (ISO 8589:2007) [35], using transparent goblet glasses, which are normally used for evaluating wines (ISO 3591:1977) [36] and are also suitable for beers. The samples were identified by a 3-digit code and presented in a randomised order to avoid the effect of the tasting order on sensory evaluation.

Each sample was poured in a quantity of approximately 25 mL and evaluated at room temperature (20 °C). All samples were tasted and judged by a trained sensory panel of 18 panellists (12 females and 6 males).

All the assessors were experts in wine sensory analysis and belonged to the scientific and technical staff of CREA (Asti). The panel was subjected to specific training on beer olfactive descriptors.

The panel ordered the samples with a ranking test (ISO 8587:2006) [37] for the overall olfactory intensity and the intensity of the odours: floral (orange blossom), citrus fruits (grapefruit), exotic (tropical) fruits (pineapple, mango, passion fruit), and vegetable (tomato leaf, fig leaf, mint, sage, rosemary, boxwood).

The same panel described the olfactory sensory profile of the samples, following a procedure derived from the ISO standards (11035:1994) [38] applied in previous studies on wine [39,40] and on beer [41]. In particular, the panel identified the odour attributes of beer samples with the help of a predefined list, already employed in a previous experience [41] with three levels of specificity, from the most generic 1st level (i.e., fruity), medium generic 2nd level (i.e., citrus, tropical, or exotic fruits), to the most specific 3rd level (i.e., lemon, grapefruit in the case of citrus; pineapple, mango, passion fruit in the case of exotic fruits). The choice of descriptors (attributes) was made based on the identification frequencies. The 2nd level odour attributes—floral, spicy, citrus, exotic fruits, and aromatic herbs—were chosen if their frequency of identification was higher than (n° assessors × n° samples/2: 18 × 8/2), and the 3rd level descriptors—orange blossom, cloves, grapefruit, caramel, honey, and sage— were chosen if their frequency was higher than (n° assessors × n° samples/4: 18 × 8/4).

The quantitative evaluations were measured twice in each beer replicate A, B, and C using unstructured scales (0–100) and in two different tasting sessions.

For data collection, the computerised data acquisition system Fizz software FZ2114 (Biosystèmes, Couternon, France) was used.

### 2.11. Statistical Analysis

The presented values are averages of biological triplicates with standard errors. The differences among the measured parameters and compounds were determined by a one-way ANOVA, followed by Tukey’s HSD test (the statistical significance level was set at *p* ≤ 0.05). Statistical analyses (ANOVA and PCA) were made using XLStat^®^ software (Addinsoft, New York, NY, USA).

The sensory ranking test results were analysed with the Friedman test and multiple comparisons (*p* ≤ 0.05). The sensory profile data were analysed with ANOVA and the Duncan test (*p* ≤ 0.05), considering the factors beer, assessor, and sensory session and their interactions.

The statistical sensory analyses were performed using XLStat^®^ software, version Sensory, 2020, 2.2.

## 3. Results and Discussion

### 3.1. Screening of β-Lyase Gene

Aromatic thiols, such as 3-mercaptohexanol (3MH) and 4-mercapto-4-methylpentan-2-one (4MMP), are potent flavour compounds that contribute significantly to the fruity and tropical aromas of beverages like beer and wine. These thiols are often present in raw materials, such as hops and grape must, in non-volatile, bound precursor forms. Their release into the final product depends on yeast enzymes, particularly β-lyase, encoded by the *IRC7* gene. This enzyme cleaves the bond between the thiol and its precursor, liberating the aromatic compound during fermentation.

The *IRC7* gene exists in two main allelic forms: a functional long form and a truncated short form. Many *Saccharomyces cerevisiae* strains, particularly those used in brewing, carry a 38-base pair deletion in *IRC7*, resulting in a premature stop codon and producing a truncated enzyme with only 340 amino acids, compared to the full-length 400 amino acids in the functional version. This mutation significantly reduces β-lyase activity and, consequently, the yeast’s ability to release thiols [27].

Yeast strains with the full-length, functional *IRC7* gene exhibit higher β-lyase activity, enabling more efficient thiol release and contributing to more intense and desirable fruity aromas in the final product. In their study, Cordente et al. [42] found that strains having the long form of *IRC7* produced about 6 and 2.5 times more 4-MMP and 3-MH compared to the amount produced by strains with the short form. For brewers and winemakers aiming to enhance thiol-derived aroma complexity, selecting strains with a functional *IRC7* allele is a key strategy. As a result, there is growing interest in the wine and brewing industries in using such strains to optimise the sensory quality of their beverages.

In this study, sixty oenological *S. cerevisiae* strains were tested by PCR assays to investigate which genotypes of the *IRC7* gene were present. As already described by [27], two sizes of PCR products were detected (Table 2). Most of the tested yeasts (56.8%) possessed the short form of the gene; 21.6% of the strains had the full-length form, and the rest were heterozygous. Of the three commercial strains tested, one had the long form and the other two were heterozygous.

These data are in agreement with the literature; in fact, several works have shown that the short form of the gene is very common in oenological yeast strains. Borneman [43] published whole-genome sequencing data from 212 strains of *S. cerevisiae*, 179 of which were oenological (94 commercial strains and 85 isolated in different parts of the world); they determined the presence of the short or long versions of *IRC7* and found that 56% of the sequenced strains were found to be homozygous for the short allele (*IRC7*S *IRC7*S), 27% were heterozygous, and only 17% were homozygous for the long form.

Recently, Riuz et al. [44] conducted a specific study on the *IRC7* gene. They investigated the distribution of this gene and its alleles in different *S. cerevisiae* populations belonging to different niches, and they observed that the great majority of wine strains were homozygous for the short allele; as a consequence of this result, they sought to investigate these data more deeply to understand the reason for this prevalence. They discovered that the *IRC7* short allele occurs together with other genes that could increase fitness and competitive advantages in wine fermentation.

### 3.2. Choice of the Strain and Evaluation of β-Lyase Activity

Since, according to the literature [27,28], strains possessing the long form of the *IRC7* gene have better β-lyase activity, the strains in this study that were shown to possess the long form of this gene in the previous screening were further tested.

S-methyl-L-cysteine is converted to methanthiol (MTL), pyruvate and ammonium through the activity of yeast β-lyase. Under analytical conditions, part of it undergoes reoxidation, dimerising into dimethyldisulphide (DMDS). In the present study, both compounds were simultaneously detected by GC-MS analysis. The strains capable of producing the highest amounts of DMDS (>2000 µg/L) were ISE 60, ISE 77, FTH, and ISE1450 (Supplementary Table S1), demonstrating good β-lyase activity. The strain ISE77 was used for fermentation trials with the two different hop varieties under study.

### 3.3. Fermentation Trials and Basic Chemical Analyses

**Figure 1:**
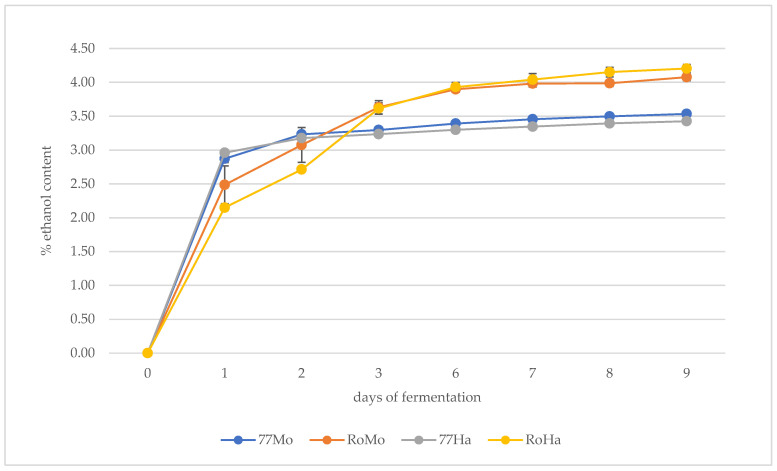
Trend of the alcoholic fermentation in the samples with the average values of three repetitions.

Figure 1 illustrates the trend of alcoholic fermentation (AF), showing the ethanol yield estimated by weight loss, produced by the yeast strains under investigation.

It can be observed that alcoholic fermentation (AF) lasted 8 days, and the commercial strain Rock reached an estimated higher final alcohol content than ISE77. From a kinetic perspective, during the initial phase of fermentation, the samples brewed with Rock yeast and HM hops exhibited a slightly slower ethanol production rate compared to the other samples. However, after three days, they reached a comparable estimated ethanol percentage to the samples fermented with Rock yeast and Mosaic^®^ hops.

All the samples were analysed for the basic chemical parameters, and the data are reported in Table 3.

As expected, the commercial strain produced a slightly higher alcoholic content than ISE77. Maltose is particularly important for fermentation completion in brewing, as it is the predominant sugar in the wort, typically present at a concentration of 50–60 g/L [45]. One of the main characteristics of brewing yeast used in the industry is its ability to consume maltose and maltotriose [46,47], which is a trait that some oenological yeasts lack.

While glucose is passively transported into the cell through facilitated diffusion, maltose and maltotriose require active transport [48]. Typically, their uptake begins only after approximately half of the wort glucose has been consumed. From our data, it is possible to affirm that the oenological strain ISE77 is suitable for beer fermentation, as it can ferment sugars contained in wort and complete alcoholic fermentation quite similarly to the commercial strain.

The total and volatile acidity data indicated that the two strains produced similar results, although the total acidity exhibited a statistically significant difference, with higher levels observed in samples fermented by ISE77.

Alcoholic fermentation is fundamental in the brewing process as it determines the beverage’s characteristic quality and sensory features [49]. Yeasts play a crucial role; the selection of an appropriate strain is largely guided by its fermentation efficiency, metabolic characteristics, and capacity to generate desirable secondary metabolites. Among these, the acidity profile contributes to shaping the overall flavour of beer [50]. The ethanol content and acidity levels observed in this study indicate that the oenological yeast performs similarly to the commercial strain, making it a promising alternative for beer production.

### 3.4. VOC and Thiol Quantification

Aromatic analyses were conducted on the 12 obtained products using GC-MS, with a particular focus on examining the volatile thiols of varietal origin.

The main results obtained are reported in Table 4.

The data were analysed statistically by considering either yeast or hops as the sole variable (see Supplementary Tables S2 and S3).

Beers fermented with different yeast strains exhibited distinct aromatic profiles. An abundant presence of fermentative compounds such as ethyl-trans-4-decenoate, isobutyl acetate, isoamyl acetate, 2-phenylethyl acetate, and phenylethyl alcohol characterises those obtained with the ISE77 yeast. The pleasant notes imparted by these molecules, often described as fruity and floral, are contrasted by the presence of 2-methoxy-4-vinylphenol, which is associated with waxy and spicy aromatic notes.

These results agree with the findings of Tocci et al. [51], where the authors explored the impact of three *S. cerevisiae* commercial strains in two different malts. They observed statistically significant differences in ester concentration, with one strain in particular producing high levels of isoamyl acetate, isopentyl hexanoate, ethyl acetate, and 2-phenylethyl acetate. The same authors noted that these findings align with literature reports identifying the aforementioned metabolites as the primary esters commonly found in beers [52,53].

The aroma profile of the beers was found to be significantly influenced by the hops used, regardless of the yeast employed. This influence is particularly evident in the presence of terpene and terpenoid compounds, which are abundant in Mosaic hops.

Myrcene represents the most abundant fraction in the extract, consistent with the findings reported in the literature [54]. Higher values of limonene, linalool, and some ethyl esters of medium-chain fatty acids were also observed. Moreover, Mosaic presented a content of citronellol 26 times higher than HM (Supplementary Table S3).

In contrast, beers hopped with HM exhibited significantly higher levels of sesquiterpenes, particularly the monocyclic humulene and γ-muurolene. The observed oil content aligns with the literature, as Hallertau Mittelfrüh has lower amounts of essential oil than the other varieties studied [55].

In recent years, several studies have highlighted the significant impact of the interaction between hops and yeast on the flavour profile of the final product. Indeed, it is acknowledged that numerous odourless aromatic precursors, primarily of thiol and terpene nature, are present in the raw materials. These precursors consist of a glycosidic unit and an aglycone, which, when released through the action of specific enzymes present in yeast, can significantly contribute to the final bouquet of the beer [31,56].

Among the bioreactions that occur during the fermentation process, one of the most impactful on the beer’s flavour profile is linked to β-glucosidase activity, which releases terpenic alcohols, responsible for very pleasant aromas such as lavender, rose, and citrus. Monoterpenic alcohols are present in hops both in free form and bound to glucosides, and their content is genotype-dependent. The hop volatile aglycones reported in the literature are linalool, β-citronellol, and phenyl ethanol. In this study, a significant interaction between yeast and hops was observed (Table 4 and Supplementary Table S2), with a 2.4-fold higher citronellol concentration when ISE77 was used instead of Rock yeast, particularly evident in the case of Mosaic hops (Table 4). It has been reported that geraniol is converted to citronellol during fermentation by *S. cerevisiae* due to the action of the enzyme exo-β-glucanase (encoded by the EXG1 gene) [56,57]. Furthermore, the results pointed out a statistically higher concentration of phenyl ethanol (accountable for floral nuances) (+69%) due to the interaction between ISE 77 and Mosaic hops in comparison to the value observed for the RoMo interaction (Table 4). Both acidic and enzymatic hydrolysis of glycosidically bound hop aroma precursors have been shown to release a range of aroma compounds, including phenyl ethanol [58].

Regarding 2-alkanones (e.g., 2-nonanone, 2-decanone), the results showed a statistically significant influence of the hops on their content (Table 4). These compounds are derived from the oxidative degradation of iso-alpha acids [59]; consistently, beers produced with Mosaic hop, which has a bitter acid content almost three times higher than Hallertau, showed significantly higher average concentrations of 2-nonanone and 2-decanone compared to those produced with Hallertau hops (+188 and +75%, respectively, see Supplementary Table S3). A slight but significant influence of the yeast strain on the concentration of these compounds in the final beers was observed. In this context, it has been reported that yeast can reduce these compounds to the corresponding 2-alkanols [60]. Finally, yeast has been demonstrated to play a pivotal role in shaping the definitive beer aroma by its capacity to physically absorb key hop-derived aromatic compounds such as α-humulene, β-caryophyllene, and β-myrcene [58]. In the present study, a significant impact of the yeast strain on the content of caryophyllene, humulene, α-calacorene, and copaene was observed, probably due to a different degree of adsorption of these molecules on the yeast cell, which may depend on the specific characteristics of the strain [61].

Table 5 presents the quantification of thiols in the analysed finished fermented beers. According to the literature, Mosaic hops are known to be rich in glutathionylated precursors of 3-MH [18]. However, in our experiment, the 3-MH content was found to be particularly high in beers brewed with HM hops, rather than those made with Mosaic hops. It is known that 3-MH can form through the biotransformation of hop-derived precursors or, to some extent, from other compounds present in the wort, such as trans-2-hexenal, a volatile compound that can act as an additional precursor for thiol production by yeast [62]. This alternative pathway should be considered in future studies to better understand the observed 3-MH levels and to determine the precise contributions of both hop-derived precursors and fermentation-related metabolic pathways to 3-MH formation.

On the other hand, higher contents of 3-MHA and 4-MMP were quantified, as expected, in the beers with Mosaic hops. Given their very low perception thresholds (4.2 and 0.8 ng/L, respectively) [63,64], these compounds play a decisive role in defining the typical aroma of beers. The fact that Mosaic contains a high content of thiols was also demonstrated by Liu et al. [18]; the authors quantified 3-MH and 4-MMP in 32 hop varieties cultivated in different countries across various harvest years and observed that the highest levels of 4-MMP were measured in Mosaic, followed by Citra.

This outcome supports the choice of the yeast in the collection, which can, therefore, be compared to the commercial strain regarding β-lyase activity, i.e., the ability to release thiols from the respective aromatic precursors in the hops.

**Figure 2:**
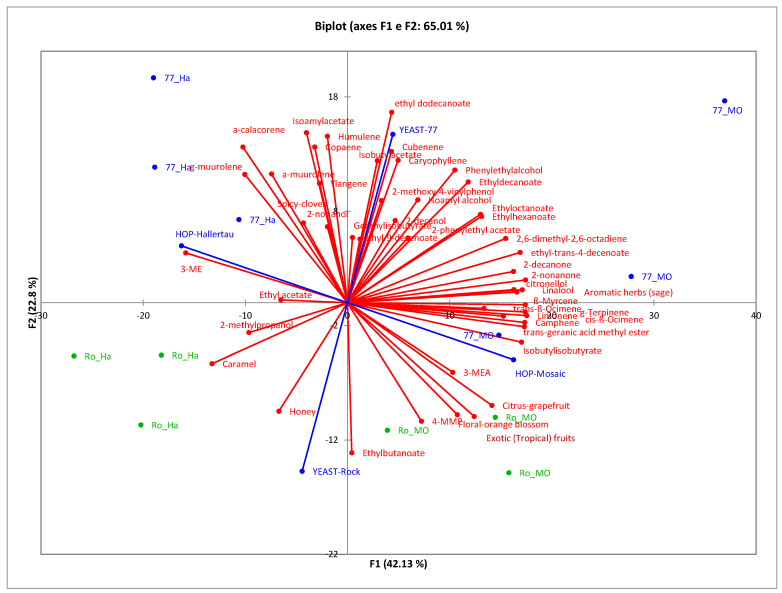
Biplot resulting from the PCA performed on the aromatic compounds quantified and the sensory descriptors of the experimental products. (red= aromatic compounds; blue: 77 strain samples; green: Rock strain samples).

Principal component analysis (PCA) was performed to visualise the relationships between yeasts, hops, and chemical compounds (Figure 2). The first two principal components, PC1 and PC2, accounted for 65.01% of the total variability. It is possible to observe that PC1 distinctly separates the hops used in this study, while PC2 separates the yeasts.

The products obtained using Mosaic^®^ are characterised by a high amount of linalool, myrcene, camphene, ocimene, trans-geranic acid methyl ester, isobutyl isobutyrate, 4MMP, and 3-MHA. In contrast, beers brewed with Hallertau hops are primarily defined by sesquiterpenes, particularly monocyclic humulene, murolene, and calacorene. Mosaic is closely linked to tropical and citrus fruit notes, supported by volatiles such as ethylbutanoate and polyfunctional thiols, while Hallertau contributes spicy and herbal characteristics, attributed to compounds like γ-muurolene and 4-vinylphenols.

Regarding the yeasts, products obtained using ISE77 are more complex and are characterised by compounds such as ethyl-trans-4-decenoate, isobutyl acetate, isoamyl acetate, and phenylethyl alcohol, which confer fruity and floral aromas. However, they also include less pleasant compounds, such as ethyl dodecanoate and 2-methoxy-4-vinylphenol. Specifically, beers fermented with ISE77, particularly those brewed with Mosaic hops (77MO), are strongly associated with fruity and floral volatiles like isoamyl acetate, ethyl dodecanoate, and phenylethyl alcohol. In contrast, 77Ha beers (those brewed with Hallertau hops) fall into the quadrant associated with spicy and clove-like aromas, while 77Mo aligns with aromatic herbal notes.

Beers fermented with the Rock yeast exhibit different profiles. The main compounds characterising these products include ethyl butanoate, which imparts a fruity aroma resembling pineapple, and 2-methylpropanol. Specifically, beers brewed with Hallertau hops (RoHa) display profiles dominated by malt-derived descriptors such as caramel and honey. On the other hand, RoMo beers (brewed with Mosaic hops) are associated with exotic fruit, citrus (grapefruit), and floral aromas, likely due to the presence of polyfunctional thiols.

### 3.5. Sensory Analysis

#### 3.5.1. Ranking Tests

**Figure 3:**
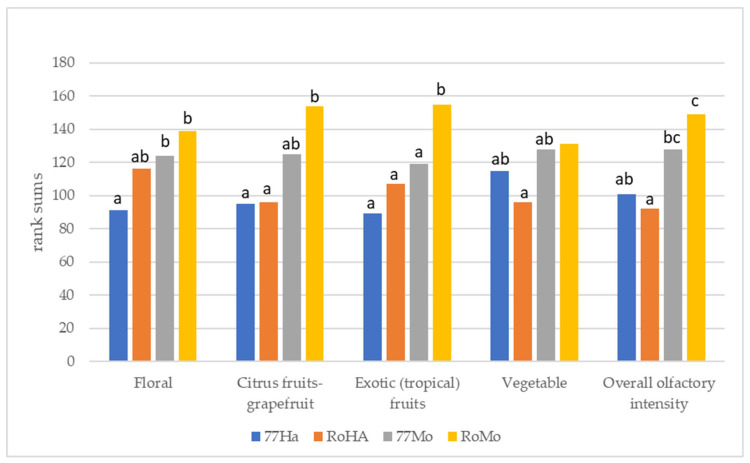
Results of the ranking tests of the three sessions carried out with the repetitions A, B, and C. Different letters indicate significant statistical differences with the Friedman test and multiple comparison test (*p* ≤ 0.05).

The results of the ranking test (Figure 3) showed significant statistical differences between the analysed samples (*p* ≤ 0.05).

The RoMo samples exhibited the highest intensity for all the parameters, while the 77Ha samples displayed the lowest intensities for floral, citrus, and exotic fruits. The 77Ha samples were very similar to the RoHa and 77Mo samples for citrus, exotic fruits, and vegetable.

The overall olfactory intensity was highest in RoMo and 77Mo samples and lowest for 77Ha and RoHa. These findings demonstrated a clear interaction between yeast and hop variety. Both yeast strains exhibited the greatest olfactory intensity in response to the Mosaic^®^ hop, while samples brewed with Hallertau hops showed the lowest intensity.

#### 3.5.2. Sensory Profiles

The panel identified seven odour attributes, which were quantified: floral (orange blossom), spicy (cloves), citrus (grapefruit), exotic (tropical) fruits, caramel, honey, and aromatic herbs (sage). The ANOVA results from the two sessions and three assay repetitions (A, B, and C) confirmed statistical differences for all these descriptors (Table 6): the factor “beer” was always significant with *p* < 0.001, except for “spicy” and “caramel”, which were significant at *p* < 0.01.

The factor “assessor” was always significant (*p* < 0.001) for the different uses of the scale. The factor “session” and the interaction “assessor x session” were never significant, except in the case of caramel, where the interaction “beer” sample x session was significant (*p* < 0.05). This attribute is less robust than the others for discriminating the beers.

The sensory olfactory profiles showed greater variability among the samples than the ranking tests, as expected. This is because the ranking test allows for a comparison of specific characteristics between samples, but not a quantitative measure of the examined characteristic.

**Figure 4:**
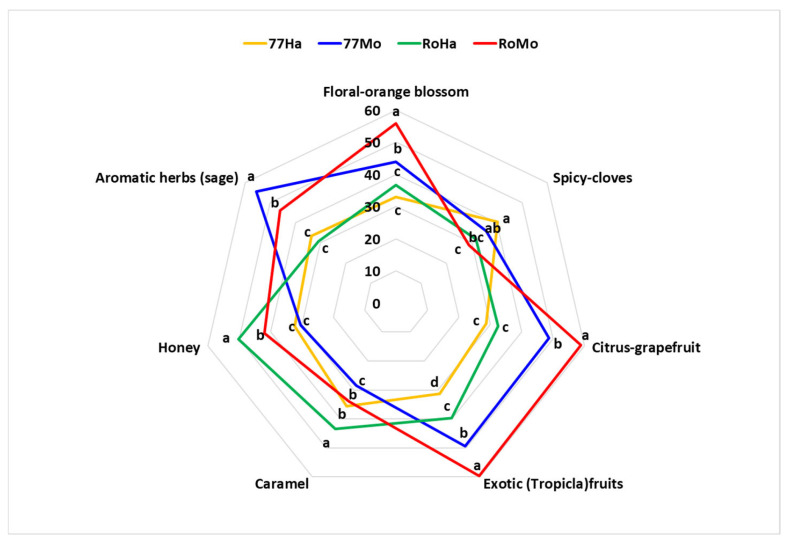
The sensory profiles of the samples. Different letters indicate significant statistical differences with ANOVA and the Duncan test (*p* ≤ 0.05).

Both yeast strains demonstrated distinctly different profiles when used with the two hops (Figure 4).

With Hallertau hops, the ISE77 strain showed similar low intensities (about 30 mm) for all the attributes, except for the higher intensities of “spicy” and “caramel”. According to [65], Hallertau hops were described as earthy, spicy, and “noble”.

The Rock strain revealed higher intensities for all the attributes, especially for “honey” and “caramel”, and lower intensities for “spicy” and “aromatic herbs”.

In the case of Mosaic^®^, samples fermented with the ISE77 strain were characterised by intense “aromatic herbs”, “citrus”, “tropical fruits” and “floral” notes. The Rock strain samples were similar for citrus and exotic fruits. However, the highest intensities were observed for “floral” and “honey”, while the lowest intensities were noted for “spicy” and “aromatic herbs”.

Both yeast strains with Hallertau showed the lowest intensities for “floral”, “citrus”, “tropical fruits” and “aromatic herbs”.

Moreover, the attribute “spicy” was highest in the samples produced with ISE77, and lower in the RoMo assays.

“Honey” and “caramel” showed the highest values with the yeast Rock, with both the hops and the lowest intensities in the samples produced with the strain ISE77.

77Mo samples exhibited the highest intensities for aromatic herbs (sage), and similar but statistically lower intensities for floral, citrus, and tropical (exotic) fruits compared to RoMo samples. Hallertau assays showed the lowest intensities for floral, citrus, and aromatic herbs. For the “tropical fruits” attribute, all the samples were statistically significantly different.

The attribute “exotic (tropical) fruits” was employed to describe beers produced with yeasts that possess the long form *IRC7* gene, in contrast with the findings of [66]. These authors reported that the sensory attributes “sweaty”, “vegetal”, and “overripe fruit” were more strongly associated with these strains than “tropical”. In the case of the ISE77 strain, other attributes were evidenced, and “aromatic herbs” was the only one attributable to “vegetal”. The others were “floral”, “spicy”, “citrus”, “caramel”, and “honey”, with different intensities depending on the type of hops. No off-odours were detected in any of the products.

## 4. Conclusions

The objective of this study was to investigate how the interplay between wine yeasts and hops during the dry hopping process influenced the chemical composition and sensory attributes of the resulting beers. Even though the same initial wort was used in all experiments, the resulting beers exhibited significant differences in aromatic compound content and sensory characteristics.

ISE77, a *Sc* strain with the long form of the *IRC7* gene and β-lyase activity, can provide interesting sensory profiles in beers using suitable hop varieties. This yeast produced beers with distinctive aromas and sensory characteristics, confirmed by the panel, and characterised by intense tropical and fruity notes, associated with passionfruit, mango, and pineapple. The characteristics observed in the beer produced with this oenological yeast are quite complex; there is an evident interaction between yeast and hop in the olfactory profile. Further assays should be done on an industrial pilot scale: conduct fermentations using the promising yeast–hop combinations identified in the study will provide valuable insights into the scalability of the process, consistency of flavour profiles across larger batches and practical challenges and solutions for implementing the recipe and to develop this strain for beer production appropriately.

These yeasts, selected for their fermentation characteristics, alcohol resistance and aroma production, can provide beer with unique organoleptic profiles. The use of oenological yeasts in brewing is a trend that allows experimentation and innovation, offering master brewers a new tool to create original and complex products, expanding the range of flavours and aromas available in the world of craft beer and responding to different consumer preferences.

A modulation in the organoleptic profile could be obtained by the accurate choice of hops. The study confirmed that Mosaic is characterised by a complex aromatic profile, described by notes of exotic and citrus fruits. In contrast, Hallertau Mittelfrüh contributes spicy and herbal characteristics. These hop varieties, when added to the brewing process, offer great potential for brewers to modify the final sensory outcome of the beer, enabling unique flavour combinations and enhancing its overall complexity.

Moreover, the described data on the interaction between hops and yeast, along with the knowledge about the evolution of aromatic compounds during fermentation, could contribute to increasing the efficacy of the brewing process.

## Figures and Tables

**Table 1:** Experimental design used in this study, with the code of the samples found in the text.

Yeast	Hop ^1^	Code
ISE77 CREA-CMVE	M	77Mo
Commercial	M	RoMo
ISE77 CREA-CMVE	HM	77Ha
Commercial	HM	RoHa

^1^ M: Mosaic^®^; HM: Hallertau Mittelfrüh.

**Table 2:** Results of the screening made by PCR on the tested *Sc* with the respective gene *IRC7* (S = short; L = long; H = heterozygote).

Yeast ISE	β-Lyase	Yeast ISE	β-Lyase	Yeast ISE	β-Lyase	Commercial	β-Lyase
2	S	102	S	579	S	FTH	L
4	S	118	H	610	S	TXL	H
9	S	120	S	652	S	STR	H
14	H	125	S	666	S		
18	S	130	S	672	S		
24	S	159	S	684	S		
27	S	165	H	689	S		
36	S	167	H	694	S		
38	H	169	H	726	S		
56	L	170	S	1085	S		
60	L	173	S	1101	S		
66	L	196	L	1216	S		
77	L	200	L	1450	L		
81	L	204	S	1480	S		
90	L	400	S	1487	S		
92	L	411	L	1490	H		
95	H	418	H	1515	S		
96	H	549	S	1521	L		
99	S	562	S	1567	H		

**Table 3:** Quantification of the basic chemical parameters. Data are means ± standard deviations of three replicates. Different letters indicate significant statistical differences (*p* ≤ 0.05, Tukey’s test).

Sample	Alcohol Content (% *v*/*v*)	Volatile Acidity g/L	Total Acidity g/L	pH
RoHa	4.177 ± 0.068 a	0.323 ± 0.025	1.633 ± 0.058 b	4.250 ± 0.010 a
RoMo	4.110 ± 0.017 a	0.340 ± 0.053	1.650 ± 0.050 b	4.243 ± 0.006 a
77Mo	3.633 ± 0.068 b	0.360 ± 0.036	1.730 ± 0.000 ab	4.217 ± 0.015 b
77Ha	3.583 ± 0.076 b	0.373 ±0.032	1.777 ± 0.040 a	4.177 ± 0.006 c

**Table 4:** Compounds identified in the volatile fraction of the beers and quantified by mass spectrometry. All data are expressed in µg/L as equivalents of the internal standard. RT: retention time of the analyte. ^§^ LRI*: linear retention index reported in the literature; ^§§^ LRI: calculated linear retention index (nc= not calculated); ^§§§^ MQ%: match quality, a percentage measure of the match between the unknown mass spectrum and the library reference spectrum. Data are means ± standard deviations of three replicates. Different letters indicate significant statistical differences (*p* ≤ 0.05, Tukey’s test).

	RT (min)	^§^ LRI*	^§§^ LRI	^§§§^ MQ%	77—Mo	RO—Mo	77—Ha	RO–Ha
Ethyl acetate	2.40	589	nc	90	357 ± 88	491 ± 59	563 ± 20	416 ± 46
2-methylpropanol	2.54	645	nc	90	100 ± 48	173 ± 42	218 ± 42	186 ± 15
Isoamyl alcohol	4.33	744	751	90	2599 ± 372	2104 ± 259	2271 ± 319	1966 ± 152
Isobutylacetate	4.40	750	778	90	88 ± 1 a	45 ± 4 b	93 ± 10 a	50 ± 5 b
Ethylbutanoate	6.51	799	798	95	45 ± 2 ab	67 ± 8 a	39 ± 3 b	64 ± 6 ab
Isoamylacetate	10.36	866	874	90	3191 ± 157 b	2176 ± 156 c	3999 ± 71 a	2737 ± 37 b
Isobutylisobutyrate	12.58	899	911	90	141 ± 4 a	118 ± 15 a	16 ± 2 b	12 ± 3 b
Camphene	14.40	954	949	81	27 ± 1 a	21 ± 2 a	4 ± 0 b	2 ± 0 b
b-Myrcene	17.94	991	986	96	13,230 ± 2078 a	9214 ± 795 a	861 ± 78 b	555 ± 99 b
Ethylhexanoate	18.46	998	999	98	1094 ± 117	922 ± 77	916 ± 51	787 ± 80
Limonene	20.12	1032	1021	99	524 ± 70 a	414 ± 47 a	78 ± 13 b	51 ± 5 b
trans-b-Ocimene	21.03	1045	1034	86	61 ± 28 a	69 ± 18 a	23 ± 9 b	14 ± 4 b
cis-b-Ocimene	21.74	1050	1045	97	157 ± 52 a	160 ± 33 a	39 ± 14 b	25 ± 7 b
g-Terpinene	22.31	1058	1044	86	41 ± 6 a	33 ± 5 a	7 ± 1 b	4 ± 0 b
2-nonanone	24.94	1091	1087	95	555 ± 62 a	386 ± 25 a	197 ± 21 b	130 ± 25 b
Linalool	25.55	1103	1099	97	1752 ± 144 a	1273 ± 142 a	725 ± 60 b	596 ± 111 b
2-nonanol	25.69	1108	1150	83	233 ± 29	227 ± 29	355 ± 215	282 ± 138
Phenylethylalcohol	26.55	1116	1110	94	2595 ± 364 a	1534 ± 158 b	2084 ± 135 ab	1367 ± 138 b
2-decanone	32.12	1172	1188	94	432 ± 41 a	295 ± 31 ab	234 ± 37 b	181 ± 32 b
Ethyloctanoate	32.77	1190	1196	97	7913 ± 1077	6401 ± 795	6270 ± 294	5008 ± 819
2-decanol	32.85	1197	1260	83	155 ± 18	141 ± 14	166 ± 17	133 ± 28
Citronellol	34.64	1228	1254	98	182 ± 12 a	75 ± 18 b	6 ± 0 c	4 ± 2 c
2-phenylethyl acetate	36.30	1270	1257	80	398 ± 172	153 ± 121	289 ± 117	117 ± 91
Vinylguaiacol	39.17	1295	1274	91	576 ± 218	162 ± 71	529 ± 220	189 ± 83
Methyl geraniate	39.75	1319	1323	97	2770 ± 129 a	2103 ± 293 a	330 ± 37 b	165 ± 32 b
n.i terpenoid	40.87	-	1404	-	100 ± 11 a	35 ± 1 b	29 ± 6 b	10 ± 5 b
a-Ylangene	41.42	1372	1368	99	30 ± 6	27 ± 10	49 ± 15	23 ± 5
a-Copaene	41.60	1380	1375	99	55 ± 20	43 ± 8	76 ± 18	53 ± 14
ethyl-trans-4-decenoate	41.88	1395	1381	95	1111 ± 194 a	657 ± 108 ab	360 ± 8 b	148 ± 51 b
ethyl-9-decenoate	42.16	1387	1389	99	465 ± 107	558 ± 44	605 ± 172	470 ± 82
Ethyldecanoate	42.51	1391	1396	99	3708 ± 747	1593 ± 229	2180 ± 808	1046 ± 379
Caryophyllene	43.14	1420	1416	99	1138 ± 336	850 ± 205	1060 ± 245	729 ± 163
Humulene	44.29	1456	1453	97	3797 ± 1230	2662 ± 543	4758 ± 1020	3430 ± 698
g-muurolene	44.95	1481	1480	98	115 ± 48	83 ± 20	234 ± 47	205 ± 54
Geranylisobutyrate	45.34	1495	1495	98	115 ± 34	60 ± 12	87 ± 45	66 ± 27
a-muurolene	45.60	1499	1509	93	67 ± 23 b	91 ± 38 ab	227 ± 43 a	77 ± 32 ab
Cubenene	46.54	1532	1552	99	27 ± 9	22 ± 2	32 ± 4	17 ± 3
a-calacorene	46.83	1538	1547	98	46 ± 13 ab	22 ± 1 b	98 ± 15 a	70 ± 10 ab
ethyl dodecanoate	48.12	1582	1596	99	746 ± 178 a	260 ± 23 b	847 ± 41 a	229 ± 40 b

**Table 5:** Average concentrations of free polyfunctional thiols (ng/L) in beer samples. Different letters indicate statistically significant differences (*p* < 0.05).

	4-MMP ^1^	3-MH ^2^	3-MHA ^3^
77Mo	54 ± 6 ab	655 ± 51 b	192 ± 10 a
RoMo	73 ± 16 a	696 ± 4 b	211 ± 10 a
77Ha	26 ± 2 b	1721 ± 162 a	88 ± 6 b
RoHa	29 ± 5 b	1539 ± 156 a	76 ± 6 b

^1^ 4-mercapto-4-methyl-2-pentanone; ^2^ 3-mercaptohexan-1-ol; ^3^ 3-mercaptohexyl acetate.

**Table 6:** ANOVA results of the sensory odour profile. Significance of F index for main effects and first-order interactions.

Attributes	Assessor	Beer Sample	Session	Assessor × Session	Assessor × Beer Sample	Beer Sample × Session
Floral (orange blossom)	***	***	ns	ns	ns	ns
Spicy (cloves)	***	**	ns	ns	ns	ns
Citrus (grapefruit)	***	***	ns	ns	ns	ns
Exotic (tropical) fruits	***	***	ns	ns	ns	ns
Caramel	***	**	ns	ns	ns	*
Honey	***	***	ns	ns	ns	ns
Aromatic herbs (sage)	***	***	ns	ns	ns	ns

*, **, ***, and ns indicate differences at *p* < 0.05 (*), *p* < 0.01 (**), and *p* < 0.001 (***) and not significant (ns), respectively.

## Data Availability

The original contributions presented in this study are included in the article/Supplementary Materials. Further inquiries can be directed to the corresponding authors.

## Supplementary Materials

The following supporting information can be downloaded at: https://www.mdpi.com/article/10.3390/foods14132357/s1, Table S1. Quantification of DMDS in the strains possessing the long form of *IRC7* gene to assess the β lyase activity; Table S2. The average values of aromatic compounds showed a statistically significant difference (*p* ≤ 0.05), with yeast being the only variable considered (*n* = 6); Table S3. The average values of aromatic compounds showed a statistically significant difference (*p* ≤ 0.05), with hop being the only variable considered (*n* = 6).
