# Supplementary material for: Exploring the Influence of Different Saccharomyces cerevisiae Strains and Hop Varieties on Beer Composition and Sensory Profiles
Source: Foods. 2025 Jul 2;14(13):2357. doi: 10.3390/foods14132357 (PMC12249449; doi:10.3390/foods14132357)
Supplement: Supplementary file 1 [file foods-14-02357-s001.zip › foods-3709372-supplementary.pdf]

Supplementary tables

Supplementary Table 1:

S1: Quantification of DMDS in the strains possessing the long form of IRC7 gene to assess the β lyase activity

SAMPLE	DMDS (GC-MS)
**1450**	+++
**1521**	+
**196**	++
**200**	-
**441**	++
**81**	+
**90**	+
**92**	+
**FHT**	+++
**56**	+
**60**	+++
**66**	++
**77**	+++
**NEGATIVE CONTROL**	-

(-) < 99 µg/L

100<(+)<999 µg/L

1000<(++)<1999 µg/L

(+++)> 2000 µg/L

Supplementary Table 2:

S2: The average values of aromatic compounds showed a statistically significant difference ($p \leq 0.05$), with yeast being the only variable considered ($n=6$).

Compounds	ISE77	Rock
Isoamyl alcohol	2436	2036
Isobutylacetate	91	48
Ethylbutanoate	42	66
Isoamylacetate	3596	2457
2-nonanone	376	259
Phenylethylalcohol	2340	1451
2-decanone	334	238
Citronellol	94	40
2-phenylethyl acetate	344	135
Vinylguaiacol	553	176
terpenoid	65	23
a -Copaene	66	48
ethyl-trans-4-decenoate	736	403
Ethyldecanoate	2945	1320
Caryophyllene	1099	790
Humulene	4278	3046
a-calacorene	72	47
ethyl dodecanoate	797	245

Supplementary Table 3:

S3: The average values of aromatic compounds showed a statistically significant difference ($p \leq 0.05$), with hop being the only variable considered ($n=6$).

Compounds	Mosaic	Ha
Isoamylacetate	2684	3369
Isobutylisobutyrate	130	15
Camphene	24	4
b-Myrcene	11222	708
Limonene	469	65
trans-b-Ocimene	66	19
cis-b-Ocimene	159	33
g-Terpinene	37	6
2-nonanone	471	164
Linalool	1513	661
2-decanone	364	208
Citronellol	129	5
Methyl geraniate	2437	248
n.i terpenoid	68	20
a -Copaene	50	65
ethyl-trans-4-decenoate	885	254
g-muurolene	99	220
a-calacorene	35	84
